# A Landmark Paper in HIV Research?

**DOI:** 10.1371/journal.pmed.0020293

**Published:** 2005-10-25

The clinical trial published in *PLoS Medicine* on whether male circumcision can prevent HIV (DOI: 10.1371/journal.pmed.0020298) is a “landmark paper” in HIV research. So said the first reviewer we sent this paper to. It is the first randomized trial to be completed of circumcision for HIV prevention, a topic that has generated a huge amount of epidemiological research and argument since the first observation that the incidence of HIV was lower in men who were circumcised. The trial was stopped at an interim analysis at the request of the trial's data safety monitoring board. At a mean of 18.1 months' follow-up there were 20 infections in the intervention (circumcised) group versus 49 in the control group, representing a protection of 60% (95% confidence interval 32%–76%).

In many ways this trial is a paradigm for the whole of HIV research; it encompasses many of the practical, social, moral, and scientific difficulties of doing such research.

The first, practical, problem of doing a trial that can test the effect of circumcision on preventing HIV infection is recruiting sufficient numbers of individuals who are prepared to be circumcised, in an area where the event rate (new infections) is likely to be sufficiently high that the trial can be done in a reasonable timeframe. It seems obvious, but perhaps not politically correct, to say that such a trial could not be done in New York or London or Paris. The trial we publish was done in South Africa, where the event rate is high and many African men now opt for circumcision in adult life by a medical practitioner.

But this circumstance leads to the second, social, problem: the ethical issues involved in doing such a trial and of applying Western standards to a non-Western setting. People in developing countries do not want to be used by Western researchers to do trials that might not be allowed in their own countries. Trials should only be done that address crucial local health problems and in a way that is appropriate for each country, and potential participants should be included in discussions about what is acceptable to them. This trial fulfills these criteria; however, HIV-positive men were not excluded from participating nor were they told of their HIV status during the trial. The authors' reasoning for not informing participants of their HIV status as a routine part of the trial (as would be likely if a similar trial could be done in a Western setting), and which was accepted by the two ethics boards that reviewed this trial, was as follows. In a country where there is stigma attached to being HIV positive, automatic exclusion of HIV-positive individuals would have potentially exposed them to discrimination. In addition, there may be benefits for HIV-positive individuals in being circumcised, including protection against other sexually transmitted diseases and against re-infection by other strains of HIV. At each visit all participants were offered advice on HIV and other sexually transmitted diseases and strongly encouraged to seek voluntary counseling and testing at a center close by where HIV status was disclosed to the patient if asked for. Although arguments rage over whether HIV status should be private or public knowledge, at the moment individuals cannot be forced into testing or indeed into knowledge of their status. Moreover, even if participants knew of their status, they would not have had access to antiretroviral drugs, as these were not available in South Africa when the trial was done. These ethical issues are discussed in two accompanying Perspectives: one by Peter Cleaton-Jones, chair of the South African ethics committee that approved the trial (DOI: 10.1371/journal.pmed.0020287), and another by Nandi Siegfried (DOI: 10.1371/journal.pmed.0020393).

The final problem that then arises is for editors: if you have any ethical questions about a study, should you publish it? This surely is what peer review and the editorial process is for. During the process of peer review here, ethical questions were raised and so also were scientific concerns. These concerns included the following: that the randomization used was unusual (but appropriate for this community); that participants were paid; that there were substantial numbers of dropouts; that the trial was stopped early (at the request of the data monitoring committee but, some might argue, too early for conclusive results); and that circumcision did appear to change the sexual behavior of participants—but in a way likely to make them more prone to infection, i.e., making the findings more robust. In considering papers for publication editors must weigh all these issues and reviewers' comments. We took particular note that this trial was approved by two experienced ethics boards.

The six reviewers and the academic editor who saw this paper were unanimous on one point: that this trial must be published, quickly. Ultimately, if these results are correct, then this is a study that offers hope. Clearly, further randomized studies will be needed to confirm the results (one in Kenya is scheduled for completion in 2007), but to not put this paper in the public domain quickly could be considered unethical in its own right.

